# Telomere length connects melanoma and glioma predispositions

**DOI:** 10.18632/aging.100935

**Published:** 2016-03-29

**Authors:** Alyson A. Endicott, Jennie W. Taylor, Kyle M. Walsh

**Affiliations:** Division of Neuroepidemiology, Department of Neurological Surgery, University of California, San Francisco, CA 94143, USA

**Keywords:** glioma, melanoma, telomere, telomerase

Glioma and melanoma are rapidly-progressing malignancies that arise from neuroectodermal origin. SEER registry data indicate that melanoma patients are at significantly increased risk of developing glioma (OR=1.42, 95%CI=1.22-1.62)[1]. Although a shared genetic etiology is suggested by melanoma-astrocytoma syndrome, an inherited cancer predisposition due to germline *CDKN2A* mutation, this Mendelian disorder cannot account for the increased co-occurrence of melanoma and glioma observed at the population level[1]. Recent epidemiologic research has identified additional germline variants that confer risk of both glioma and melanoma and which implicate telomere maintenance in the development of these cancers.

Telomeres are repetitive DNA sequences that cap and protect chromosomes and are depleted with each somatic cellular division. Because telomere attrition causes replicative senescence, increased telomere length may allow for prolonged cell survival, increased accrual of mutations, and greater propensity for malignant transformation. A very large genome-wide association study (GWAS) conducted by the ENGAGE Consortium has identified seven genes that are reproducibly associated with inter-individual variation in leukocyte telomere length (LTL), including single nucleotide polymorphisms (SNPs) in: *ACYP2, TERC, NAF1, TERT, OBFC1, ZNF208,* and *RTEL1*[2]. In addition to the effects of these genes on LTL, recent GWAS also identified glioma susceptibility loci near *TERT*, *TERC*, and *RTEL1*[3] and melanoma susceptibility loci near *TERC*, *TERT*, *OBFC1,* and *RTEL1*[4]. Taken together, these GWAS suggest that telomere length may be a common link between the genetic architecture of melanoma and glioma predisposition.

In a recent Mendelian randomization study [5], Walsh *et al*. assessed the impact of LTL on glioma risk in independent case-control datasets from the UCSF Adult Glioma Study (652 patients, 3735 controls) and The Cancer Genome Atlas (478 patients, 2559 controls). A genetic score predictive of LTL was constructed using a weighted linear combination of subject genotype at the seven lead LTL-associated SNPs first identified by the ENGAGE Consortium [2]. A genetic predisposition to longer LTL was associated with increased glioma risk in both the discovery (7.8×10^−8^) and replication sets (1.5×10^−3^), and glioma risk increased monotonically with increasing septiles of LTL[5]. In analyses of individual LTL-associated SNPs, those in *TERC*, *TERT* and *OBFC1* were significantly associated with glioma risk [5]. By using inherited genetic variants, present since birth, to estimate inter-individual differences in telomere length, associations between telomere length and glioma risk could not be confounded by age, environmental factors, chemotherapy or tumor microenvironment.

In another recent Mendelian randomization study, Iles et al. investigated the effect of telomere length on melanoma risk using the same seven SNPs (11108 patients, 13933 controls) [6]. They too constructed a genetic score predictive of LTL and found that higher telomere scores were strongly associated with increased melanoma risk (P=8.9×10^−9^)[6]. Additionally, grouping scores into quartiles revealed a significant linear trend between increasing LTL and increasing melanoma risk. In analyses of individual LTL-associated SNPs, those in *TERC*, *TERT*, *NAF1*, and *OBFC1* were significantly associated with melanoma risk [6].

**Figure 1:**
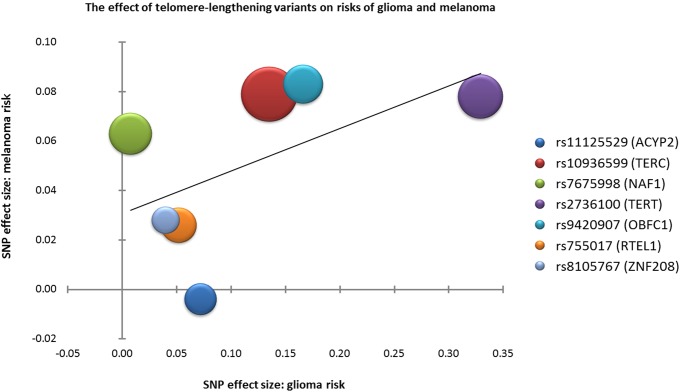
The effect of telomere-lengthening variants on the risks of glioma and melanoma Seven single nucleotide polymorphisms (SNPs) associated with inter-individual variation in leukocyte telomere length (LTL) were evaluated for their association with risks of glioma [5] and melanoma[6]. The x-axis plots the effect size of each SNP on glioma risk, while the y-axis plots the effect size of each SNP on melanoma risk. Effect sizes are displayed as Beta values from ancestry-adjusted logistic regression analyses. A positive correlation between a SNP's effect on glioma and its effect on melanoma was observed (r=0.56). Bubble sizes are proportional to the effect size of each SNP on LTL[2]. Beta values for glioma and melanoma are for each additional copy of the allele associated with longer LTL.

Because the Mendelian randomization studies of glioma [5] and of melanoma [6] investigated the same seven LTL-associated SNPs, we can directly compare the effect that these SNPs have on both cancer types. As seen in Figure 1, a positive correlation between a SNP's effect on glioma risk and its effect on melanoma risk is observed (r=0.56). The strongest effects were observed at rs10936599 (*TERC*), rs2736100 (*TERT*) and rs9420907 (*OBFC1*). A strong melanoma association was also detected at rs7675998 (*NAF1*), but a similar association was not observed with glioma. Correspondingly, SNPs in the telomerase component genes *TERC* and *TERT* also had the strongest association with LTL in the ENGAGE Consortium data, followed by those in *NAF1* and *OBFC1*.

Although GWAS are designed to detect common low-penetrance risk alleles, next-generation sequencing approaches can address the impact of rare variation on cancer risk. Through whole-exome sequencing of families with multiple glioma diagnoses, mutations in *POT1* (*Protection of Telomeres 1*) have been identified as high-penetrance glioma risk factors. *POT1* encodes one of six members of the shelterin complex, playing a key role in telomere protection and telomerase regulation. Remarkably, high-penetrance germline mutations in three shelterin complex genes (*POT1*, *ACD*, and *TERF2IP*) have also been identified as causes of familial melanoma [7]. Thus, rare variants in shelterin complex genes link melanoma and glioma predisposition to additional Mendelian disorders, the first since *CDKN2A* deletions were characterized in melanoma-astrocytoma syndrome.

The convergence of melanoma and glioma predisposition on genes involved in telomere maintenance strengthens the connection between these malignancies. From a clinical perspective, neuro-oncologists may want to consider the potential benefits of regular skin checks for their glioma patients. This is increasingly important as adults with lower-grade gliomas are surviving longer after diagnosis (>10 years). As future research begins to identify the full complement of genes involved in telomere lengthening, we may discover more shared genetic etiology linking these malignancies.
